# Birth outcomes among Métis women and infants in Manitoba, Canada: A linked administrative data study using health system and justice system data.

**DOI:** 10.23889/ijpds.v7i3.1927

**Published:** 2022-08-25

**Authors:** Jennifer Enns, Julianne Sanguins, Randy Walld, Marni Brownell, Lorna Turnbull, Marcelo Urquia, Alyson Mahar, Farzana Quddus, Hygiea Casiano, Shannon Allard Chartrand, Nathan Nickel

**Affiliations:** 1 University of Manitoba; 2 Manitoba Métis Federation; 3 Manitoba Centre for Health Policy, University of Manitoba

## Objectives

Manitoba has one of the highest incarceration rates among Canadian provinces, and because of Canada’s racist and colonial policies, the number of Métis women in custody is disproportionately high. In this study, we examined birth outcomes among Métis and all other Manitoba women who were incarcerated while pregnant.

## Approach

Using linked administrative health and justice system data, we developed a 2004-2017 cohort of mother-infant dyads with a live birth (n= mothers; n= infants). We identified all Métis mother-infant dyads, and then divided them into 3 mutually exclusive groups: a) prenatal incarceration; b) post-natal incarceration; c) no incarceration. We developed matched comparison groups for each exposure based on Métis identity, maternal age, geography and income, and, after adjusting for other potentially confounding variables (e.g., maternal age, maternal mental health, and socioeconomic factors), we examined risk differences in birth outcomes among Métis women and infants between exposure groups.

## Results

The cohort included n=534 mothers who were incarcerated while pregnant (a), n=1,677 mothers incarcerated postnatally (b), and n=14,084 mothers never incarcerated (c). When we compared prenatal vs postnatal incarceration (a vs b), we found that women incarcerated during the prenatal period were more likely to have a low birth weight infant (RD: 4.2, 95% CI: 3.0, 5.4), preterm birth (RD: 5.1, 95% CI: 3.7, 6.5) or Caesarean birth (RD: 5.5, 95% CI: 3.8, 7.1) and were less likely to have a large-for-gestational-age infant (RD: -3.8, 95% CI: -5.1, -2.5) or to initiate breast feeding (-3.4, 95% CI: -5.5, -1.3). In the prenatal vs no incarceration comparison (a vs c), the same outcomes were statistically significant as above; however, the risk differences were even more pronounced.

## Conclusion

Prenatal incarceration is associated with poor birth outcomes for all mothers and infants in Manitoba; however, Métis women and children are disproportionately affected, further perpetuating inequities they may already experience. Canadians must acknowledge the harms of racist policies and practices, and work to support the health and well-being of Métis people.

